# Biomass-Derived Carbon-Based Electrodes for Electrochemical Sensing: A Review

**DOI:** 10.3390/mi14091688

**Published:** 2023-08-29

**Authors:** Christian Onfray, Abdoulaye Thiam

**Affiliations:** Programa Institucional de Fomento a la Investigación, Desarrollo e Innovación, Universidad Tecnológica Metropolitana, Santiago 8940577, Chile

**Keywords:** biomass waste, carbon-based electrode, circular economy, green synthesis, low-cost catalyst

## Abstract

The diverse composition of biomass waste, with its varied chemical compounds of origin, holds substantial potential in developing low-cost carbon-based materials for electrochemical sensing applications across a wide range of compounds, including pharmaceuticals, dyes, and heavy metals. This review highlights the latest developments and explores the potential of these sustainable electrodes in electrochemical sensing. Using biomass sources, these electrodes offer a renewable and cost-effective route to fabricate carbon-based sensors. The carbonization process yields highly porous materials with large surface areas, providing a wide variety of functional groups and abundant active sites for analyte adsorption, thereby enhancing sensor sensitivity. The review classifies, summarizes, and analyses different treatments and synthesis of biomass-derived carbon materials from different sources, such as herbaceous, wood, animal and human wastes, and aquatic and industrial waste, used for the construction of electrochemical sensors over the last five years. Moreover, this review highlights various aspects including the source, synthesis parameters, strategies for improving their sensing activity, morphology, structure, and functional group contributions. Overall, this comprehensive review sheds light on the immense potential of biomass-derived carbon-based electrodes, encouraging further research to optimize their properties and advance their integration into practical electrochemical sensing devices.

## 1. Introduction

Biomass (BM) refers to a diverse range of biogenic organic and inorganic solid products formed through natural and anthropogenic processes. It includes natural constituents originating from land and water vegetation, as well as from waste materials resulting from animal and human food digestion. Additionally, BM includes technogenic by-products derived from processing various natural resources. With the global population’s ever-increasing demand for food, technology, energy, and essential resources, coupled with the intensification of agricultural, industrial, and forestry activities, vast quantities of biomass wastes are generated annually.

Biomass, as a renewable resource, exhibits remarkable potential in the development of cost-effective materials with high activity for various applications, including energy production, energy storage, H_2_ storage, catalysis, adsorption, wastewater treatment, CO_2_ capture, solar cells, drug delivery, and sensing [1,2,3,4]. Generally, biomass comprises three major components: hemicellulose, cellulose, and lignin. However, the chemical composition of biomass varies significantly due to its wide variety and quantity, primarily influenced by the source and treatment methods employed for its conversion. The classification most used for sorting biomass waste is related to their origin in nature, such as (i) herbaceous biomass, (ii) woody biomass, (iii) animal and human waste, (iv) aquatic biomass, (v) fungi-derived biomass, and (vi) industrial waste, all of which can also be divided into several subgroups. The chemical compositions of the main groups are tabulated in Table 1.

The composition of naturally derived biomass is intricately influenced by various factors, including the species and specific part of the trees or plants, their growing conditions (such as sunlight, geographic location, climate, seasons, soil types, water availability, pH, and nutrient content), and potential contamination from pollution, fertilizer, and pesticide usage, harvesting time, and collection technique, among many others [6]. Besides the significant presence of C, H, and O, biomass residues also contain smaller concentrations of elements such as S and N. Additionally, trace amounts of Al, Mn, Na, Si, Ca, Cl, Fe, K, Mg, and P have been detected in biomass residues.

In general, naturally derived biomass has been highlighted due to its excellent properties, such as thermal stability, mechanical strength, crystallinity, and surface modifiability, which allows the modulation of the properties according to the application of interest [3]. In contrast to the uniform and well-controlled structure commonly observed in traditional materials, biomass materials are conformed by natural uniformed structures, which reach down to the nanoscale. These inherent characteristics confer interesting properties to the prepared materials, including good mechanical and electrochemical properties. Notably, a strong relationship between the structure of porous carbon materials and their electrochemical properties has been established. The porous structure usually goes hand-in-hand with a large surface area, which allows the proper diffusion of the analyte molecules to and from pores over the whole surface, considerably improving the performance of the electrochemical sensor. This relation empowers the rational design of new biomass-derived porous carbon materials for electrochemical applications, including their applications in supercapacitors, batteries, and electrochemical sensors [5].

Several materials, such as metallic materials (Hg, Pt, Au, Ag, stainless steel), metal oxides (iron oxides, manganese oxide, titanium oxide, cobalt oxide), carbon-based (glassy carbon, graphite, carbon black, fullerenes, carbon nanotubes, graphene-related materials), hybrid materials, metal–organic frameworks (MOFs), organic polymers, and different nanomaterials, have been studied over the last decades to prepare electrochemical sensors with good detection performance. Regarding this, carbon-based materials have become popular because they have been shown to be good candidates for electrochemical sensing. Carbon-based materials have relatively inert electrochemistry and high electrochemical activity for many redox reactions, their surface can be easily modified, and they are relatively cheap. In this context, biomass-derived carbon-based materials have become more popular over recent years due to their abundant precursors in the natural environment and the easier synthesis methods, which considerably reduce the cost of fabrication of novel electrochemical sensors. Furthermore, the use of biomass-derived carbon-based materials for electrochemical applications not only allows the possibility of fabricating rapid, sensitive, and robust sensors, but also allows the reuse of the wastes generated by the world’s needs and the evolution of technology. This permits sustainable practice in sensing and developing a circular economy.

Biomass-derived carbon materials have been synthesized for many applications in different dimensions and sizes, which, together with some other features such as surface area, porosity, conductivity, and stability, determine the sensor performance. The most common classification is related to their dimensions:

- Zero-dimensional (0D): These are monodispersed spherical nanoparticles with an average size of less than 10 nm, composed of a carbon-based core with several functional groups attached to the surface (such as hydroxyl, carboxyl, and amino groups [7]. Moreover, 0D carbons, such as carbon quantum dots and fullerenes, have been studied in different fields.
- One-dimensional (1D): This category includes 1D biomass materials with a fibrous or tubular structure.
- Two-dimensional (2D): These carbonaceous materials exhibit sp^2^ hybridization, offering high energy storage and conversion potential due to their abundant active surface edges and strong in-plane covalent bonding.
- Three-dimensional (3D): Biomass structures in 3D form possess well-connected and large pores, facilitating a continuous electron channel for efficient electrical contact, thereby accelerating the ion transfer.

The following review classifies, summarizes, and analyses different research made over the last 5 years, including the main methodologies for the development of new carbon-based materials for electrochemical sensing using biomass and biomass-derived wastes as precursors. The review also highlights the different sources, such as herbaceous, wood, animal and human wastes, aquatic, and industrial waste, along with several strategies made for the modulation of their physicochemical properties to enhance their sensing performance. A discussion of several analytical parameters using different materials is also presented.

## 2. Synthesis Methods

**Figure 1:**
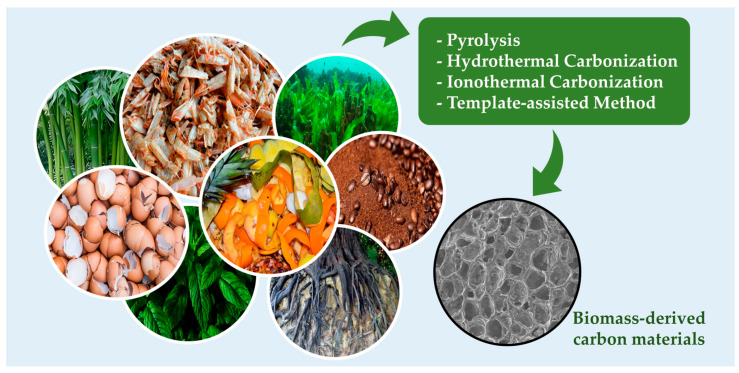
Main synthesis methods for preparing biomass-derived carbon materials for sensing applications.

Biomass-derived carbon materials can be prepared using various methods depending on the desired application and the type of biomass required; however, the most commonly employed techniques for electrochemical applications are described below and illustrated in Figure 1. The synthesis method and associated parameters, such as temperature, pressure, residence time, pH, gas atmosphere, solvent, and substrate concentration, play a pivotal role in determining the structure and activity of the resulting material. Carefully controlling these synthesis parameters is crucial in tailoring the carbon material’s properties to meet specific electrochemical requirements [8,9].

### 2.1. Pyrolysis of Biomass

The method most commonly employed for obtaining carbon materials from biomass is pyrolysis. In this process, carbon materials are heated at high temperatures in the presence of an inert atmosphere, leading to the formation of liquid (bio-oil), solid (which is frequently known as charcoal or char), and gaseous (combustible gas) products. During pyrolysis, when samples are heated, the moisture becomes volatilized, eliminating gases like CO_2_, CH_4_, and CO. Subsequently, hemicellulose undergoes degradation at temperatures ranging from 220 to 315 °C, followed by the pyrolysis of the cellulose and lignin at higher temperatures. This process generates single and multi-phenolic molecules, which then polymerize and recondense. The structure rearranges through cross-linking, leading to turbostratic graphene sheets [10]. By carefully adjusting the temperature and atmosphere and incorporating different catalysts, the morphology and physicochemical properties of the pyrolyzed materials can be effectively modulated. [11].

### 2.2. Hydrothermal Carbonization

Hydrothermal carbonization (HTC) is a widely utilized thermochemical method for synthesizing porous carbon materials. It finds broad applications with carbohydrates, agricultural waste, forest by-products, and crude plant materials. In this process, precursors are heated at lower temperatures than pyrolysis (180–250 °C) under autogenous pressure maintained for a specific time. When the temperature is increased (>374 °C), the hydrogen bonds of water are weakened, generating its dissociation into acidic hydronium ions (H_3_O^+^) and basic hydroxide ions (^−^OH). The generation of hydronium ions leads to acid-catalyzed reactions of organic compounds, generating diverse reactions, such as hydrolysis, dehydration, decarboxylation, polymerization, and aromatization. In this treatment, the hydrolysis process is followed by polymerization and the formation of spherical particles. HTC is categorized into four main groups based on operating conditions: High-temperature HTC, Low-temperature HTC, Microwave-assisted HTC, and Additive-assisted HTC [8,12]. Each variant offers unique advantages, making HTC a versatile and valuable technique for producing various porous carbon materials.

### 2.3. Ionothermal Carbonization

This method utilizes ionic liquids as crucial components, owing to their exceptional thermal stability, low solvent volatility, and high solubility with biomass. In this method, the ionic liquid is used for the solvent and template, which ideally eliminates the competition between solvent–catalyst and template–catalyst. This technique yields porous carbon materials with noteworthy attributes, including high surface area, excellent heteroatom doping, well-defined micro- and mesoporous distribution, and high carbonization yields. These characteristics contribute to improved ion transport and more active sites, making ITC a promising approach for synthesizing advanced porous carbon materials [13].

### 2.4. Template-Assisted Method

This method is one of the most effective approaches for crafting porous carbonaceous materials with a precisely controlled structure and morphology. Utilizing templates guides the material’s growth during the process, enabling the creation of hollow microporous structures that offer a multitude of active sites and a high surface area, enhancing the physicochemical properties and yielding materials with higher electrochemical activity. Template-assisted methods can be classified into two groups: hard template-assisted synthesis and soft template synthesis. In the former, the hard template is challenging to remove; instead, in the soft template synthesis, the template can be easily removed after the carbonization step [14].

The advantages and disadvantages of the different methods are summarized in Table 2. While all of the mentioned methods have been employed, pyrolysis and hydrothermal carbonization stand out as the most widely used techniques for synthesizing biomass-derived carbon materials in developing electrochemical sensors. A notable drawback of the pyrolysis method is the limited variety of functional groups on the material’s surface. However, to enhance the electrocatalytic properties, porosity, and specific structural features of the prepared materials, researchers have implemented various experimental strategies, as discussed in the following section.

## 3. Activity Enhancement Strategies

In the last decade, different attempts have been made to enhance the electrocatalytic activity of the existing biomass-derived carbon-based materials, leading to the exploration of various methods. The main strategies for this enhancement aim to increase the surface area by creating a surface with higher porosity, higher pore volume, and a higher number of active sites, along with doping with different features into the carbon-based structure. These modifications provide a higher electrocatalytic activity and adsorption capacity to the biomass-derived carbon-based catalyst. The following section discusses the most used strategies.

### 3.1. Activation Process

**Figure 2:**
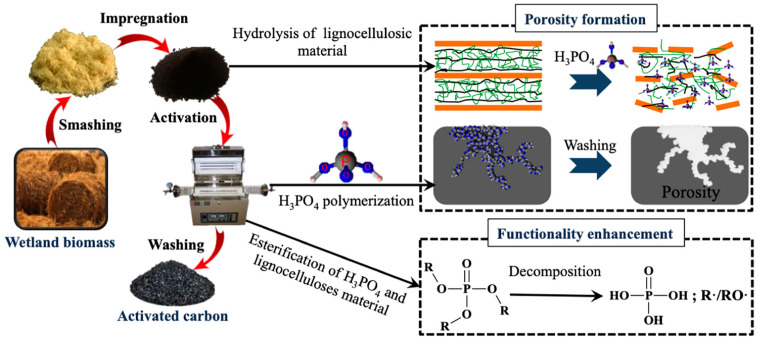
Scheme of activated carbon production from wetland biomass with conventional H_3_PO_4_ activation [20].

The most commonly used strategy to enhance the activity of carbon-based materials is the activation process, which involves physical, chemical, and/or microbial treatments. This process aims to optimize the material’s porous structure and modify the functional groups on the catalyst surface. Within the activation process, several methods stand out and are described below. The surface oxidation method utilizes temperature (>400 °C) and oxidants, such as HNO_3_, H_2_O_2_, KMnO_4_, H_2_SO_4_, and H_3_PO_4_, capable of hydrolyzing the carbon-based materials (Figure 2) and modifying the functional groups of its surface, achieving abundant oxygen-containing acid functional groups (e.g., carbonyl, carboxyl, ester, cyano groups) [15]. The surface can also be modified using temperature (>400 °C) and reducing agents, such as N_2_, H_2_, NH_3_, and some alkali salts, such as NaOH and KOH, achieving a surface with alkaline functional groups and with an increased non-polarity [16,17,18,19,20]. Some other salts, such as ZnCl_2_, FeCl_3_, and K_2_CO_3_, have also been used. These treatments are usually performed to activate biomass materials containing cellulose, such as wood, sawdust, and fruit pits. Some operational variables that affect the characteristics and properties of the final product are the activator agent used, the amount of impregnation, the ratio, and the heating temperature. The main disadvantage of this method is the long washing step to remove the activator agent from the final mixture at the end of the activation process, besides the contaminated wastewater produced during this washing step [21].

### 3.2. Heteroatom Doping

**Figure 3:**
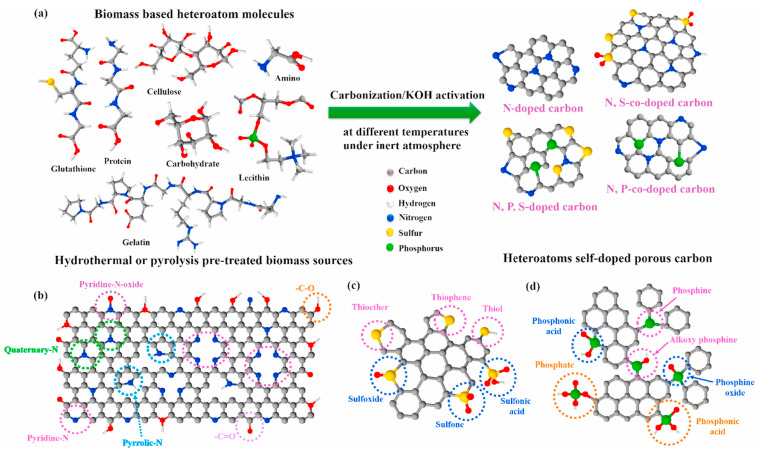
(**a**) Schematic illustration of heteroatoms self-doped porous carbon derived from biomass sources; types of (**b**) nitrogen, (**c**) sulfur, and (**d**) phosphorous [23].

Biomass-derived carbon materials are commonly doped with heteroatoms like B, N, P, and S. These heteroatoms are introduced into the carbon network of different molecules (Figure 3a) when the precursors are heated, developing a catalyst with a synergic effect on the carbon materials. This doping enhances their chemical and physicochemical properties, improving electrochemical sensing capabilities [22].

Nitrogen is the most common heteroatom used due many reasons, mainly due to he following reasons: (a) its number of electrons can be easily tuned with C when doping; (b) N acts as an electron donor, giving more electrons to the delocalized carbon network, leading to an increase in electric conductivity; (c) the atomic radius of N is similar to C, which reduces the lattice mismatching. When N bonds to the sp^2^ carbon network, different N-sites are formed, such as N-pyridine, N-pyrrolic, N-graphitic, and N-oxidized pyridine (Figure 3b), which all possess different activities. Sulfur has similar functional groups to oxygen (thiols, sulfides, disulfides). However, when S is introduced into the carbon network, its larger radius results in the disruption of the planar structure, creating defects that act as active sites for different redox reactions, and different sites such as thiophene, thioether, sulfoxide, sulfone, and sulfonic acid are formed (Figure 3c). Besides this, sulfur enhances the capacitive performance of the carbon framework due to the location of higher electron density at the surface of C due to its synergistic activation with the electron-rich sulfur. Phosphorus is an n-dopant element, which possesses the same doping characteristics of N, but possesses a larger radius of 0.110 nm than N (0.070 nm) and S (0.104 nm), resulting in a larger interlayer spacing. Moreover, P atoms regularly exhibit an sp^3^ configuration, which causes distortions and an open-edge morphology of the carbon network, providing more active sites. Furthermore, P-doping introduces many P-O groups (Figure 3d), such as phosphate, phosphine, and phosphine oxide, on the carbon surface, improving its electrochemical properties.

### 3.3. Other Activity Enhancement Strategies

Along with the activation of the surface and the heteroatom doping, introducing other features into the carbon network has shown to improve the activity of the material. On the one hand, the introduction of metal sulfide into the carbonaceous materials enhances their affinity for target molecules due to the strong metal sulfide bond, facilitating binding interactions. This strategy also increases the effective surface area, creating more active sites and promoting electrolyte penetration and ion diffusion within the porous structure [24]. On the other hand, the introduction of metal, metal alloy, and metal oxide nanoparticles into the carbon structure has demonstrated superior catalytic performance, rapid mass transport, a high electroactive area, improved electrode–electrolyte interface, and enhanced electron transfer kinetics [25].

## 4. Classification and Applications

### 4.1. Herbaceous Biomass and Derivates

Herbaceous biomass comprises a variety of plant forms, including grasses, flowers, stems, grains, seeds, leaves, roots, and fruit wastes. A significant amount of this waste comes from agricultural activities and by-products from the food, fiber, and agro-industries. Due to its abundance, cost-effectiveness, and ease of processing, herbaceous biomass and its derivatives are widely used for various applications [26].

#### 4.1.1. Fruit-Derived Biomass

**Figure 4:**
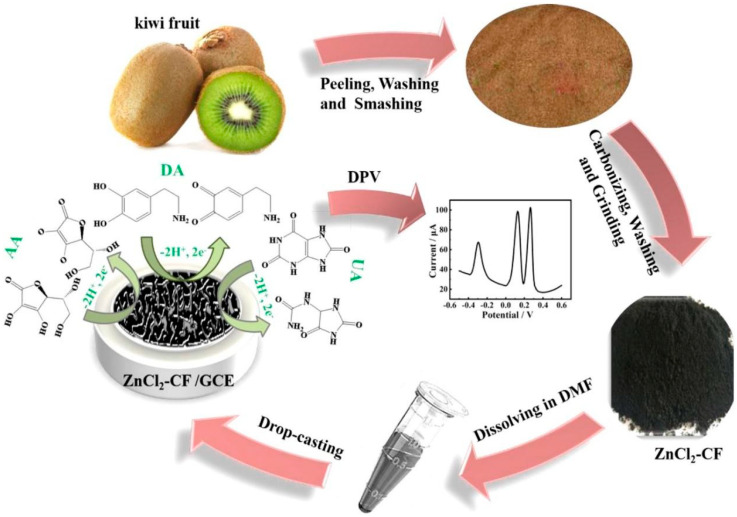
Schematic methodology for preparing the electrochemical sensor using kiwi peel [39].

Numerous fruit wastes, such as peels and husks from fruits like apples, bananas, oranges, pomelo, and dragon fruit, have been extensively studied for their potential to develop highly active electrochemical sensors [27,28,29,30,31,32,33,34,35,36,37,38]. In a study by Zhang et al. [39], an electrochemical catalyst was fabricated to simultaneously detect ascorbic acid, dopamine, and uric acid using differential pulse voltammetry (DPV) analysis. The authors prepared the catalyst by combining kiwi skin and zinc chloride nanoparticles (used as an activator) and subjected the mixture to pyrolysis treatment at 800 °C under an argon atmosphere. The catalyst exhibited a regular, uniformly distributed nanoporous structure with a high Brunauer, Emmett, Teller (BET) surface area ~1226 m^2^/g) and high pore volume (1.14296 cm^3^/g). The resulting sensor platform was created by modifying glassy carbon electrodes through drop-coating with the prepared catalyst (Figure 4). The constructed electrode showed a high surface area with micro and mesoporous structures and abundant edges and defects, which significantly enhanced contact with the active sites and facilitated redox reactions. Under optimized conditions, the system demonstrated remarkable sensitivity and selective response in the coexistence of ascorbic acid, dopamine, and uric acid. It achieved linear response ranges of 0.05–200 µM, 2–2000 µM, and 1–2500 µM, respectively, and detection limits (LODs) of 0.02 µM, 0.16 µM, and 0.11 µM, respectively. The excellent sensing performance was attributed to the abundant edges and defects of the surface of the prepared catalyst, which facilitate the electrolyte penetration and transportation, and to the high contact probability between the reactant molecules and the active sites.

Recently, El Hamdouni et al. [40] developed an eco-friendly walnut shell biochar for the electrochemical simultaneous detection of different heavy metal ions (Cd^2+^, Pb^2+^, Cu^2+^, Hg^2+^) in water and soil by square-wave voltammetry (SWV). The catalyst was prepared by pyrolysis of walnut shell at 600 °C for 2 h under vacuum. Subsequently, the carbon paste electrode was prepared by mixing the walnut shell biochar with graphite, which was then covered on the surface with poly-tyrosine by electrodeposition. The covering introduced many -OH and -COOH groups to the surface, constituting the possible binding sites for positively charged analytes. Electrochemical characterization demonstrated that the prepared electrode exhibited a synergistic effect between p-tyrosine and biochar, resulting in an increased electroactive surface area and improved electrical properties of the electrode. Consequently, the sensor displayed excellent sensitivity towards heavy metal ions, achieving low LODs close to 0.0001, 0.0002, 0.0002, and 0.0004 µM for Cd^2+^, Pb^2+^, Cu^2+^, and Hg^2+^, respectively. The development of this eco-friendly sensor using walnut shell biochar demonstrates its potential as a cost-effective and efficient material for the electrochemical detection of heavy metal ions in environmental samples.

#### 4.1.2. Plant- and Leaf-Derived Biomass

The unique chemical composition and three-dimensional structures of plant- and leaf-derived biomass provide ample opportunities for engineering novel electrode materials with tailored properties. These properties include large surface areas, hierarchical porous structures, and the ability to capture and interact with analytes of interest, resulting in improved electrochemical sensing performance [41,42,43,44,45,46,47,48,49,50]. Manickaraj et al. [51] successfully developed a highly porous houseplant (*Sansevieria trifasciata)* biomass-derived activated carbon using a supercritical CO_2_ route. The catalyst was fabricated for Metol electrochemical sensing, utilizing a modified screen-printed carbon electrode by DPV analysis. The fabrication of the SC-ST-AC catalyst involved a thermal and activation process. The biomass was pre-carbonized at 400 °C for 4 h under an N_2_ atmosphere, followed by a chemical activation process using a 3 M KOH solution for 12 h. Subsequently, the sample was subjected to the CO_2_ process and was further heated at 600 °C for 2 h under a N_2_ atmosphere. The prepared catalyst exhibited higher porous architecture and superior phase purity with an amorphous nature compared to the catalyst obtained for a conventional method. Moreover, the process not only facilitated the porous nature, but also enhanced the activation reaction kinetics, contributing to its enhanced electrochemical performance. The presence of the porous nature on the carbon surface was attributed to the reaction of K^+^ from KOH with the carbon on the biomass-derived surface (1)–(6).

6KOH + 2C → 2K + 3H_2_ + 2K_2_CO_3_ (1)

K_2_CO_3_ + C → K_2_O + 2CO (2)

K_2_CO_3_ → K_2_O + CO_2_ (3)

CO_2_ + C → 2CO (4)

K_2_CO_2_ → K_2_O + CO (5)

K_2_O + C → 2K + CO (6)

**Figure 5:**
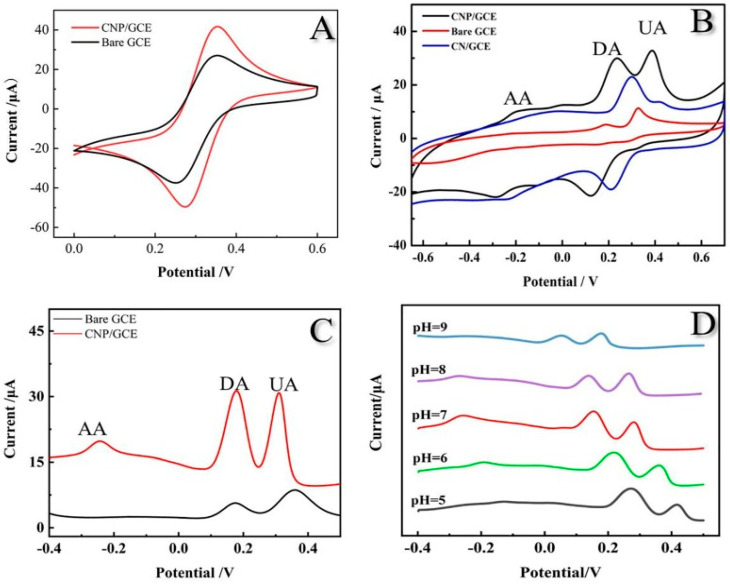
(**A**) Cyclic voltammogram of different electrodes in a solution of 0.1 M KCl containing 5.0 mM Fe(CN)_6_^3−^/^4−^; (**B**) cyclic voltammogram of bare glassy carbon electrode, CN-modified glassy carbon electrode, and phosphorous-doped nitrogenous porous carbon material modified glassy carbon electrode in 0.1 M phosphate buffer solution (pH 7.0) with 0.2 mΜ ascorbic acid, DA, and UA; (**C**) DPV phosphorous-doped nitrogenous porous carbon material-modified glassy carbon electrode, and bare glassy carbon electrode in 0.1 M phosphate buffer solution (pH 7.0) with 0.2 mΜ ascorbic acid, DA, and UA; (**D**) effect of pH value [52].

Furthermore, the optimized catalyst exhibited a superior detection limit (0.005 µM L^−1^) and sensitivity (0.854 µA µM^−1^ cm^−2^), which were attributed to the higher porous, active sites, and charge transfer efficiency of the prepared catalyst. The development of this highly porous biomass-derived activated carbon presents a promising approach for efficient electrochemical sensing applications, with potential implications in various analytical and environmental monitoring systems. Similarly, a cost-effective, green, and environmentally friendly phosphorous-doped nitrogenous porous carbon material was fabricated by Huang et al. [52] using lotus leaves for the electrochemical and simultaneous sensing of ascorbic acid, dopamine, and uric acid. The prepared catalyst showed a high BET surface area and mesoporous structure, facilitating low resistance channels and providing more active sites, thereby enhancing the electrochemical sensing performance. Electrochemical characterization revealed that the phosphorous-doped nitrogenous porous carbon material-modified glassy carbon electrode improved the conductivity and promoted electron transmission compared to the bare glassy carbon electrode (Figure 5A). Moreover, the prepared electrode demonstrated distinct and higher oxidation peak currents for ascorbic acid, DA, and UA using cyclic voltammetry (Figure 5B) and DPV (Figure 5C) compared with the bare glassy carbon electrode. The authors attributed this improvement to two potential factors: (i) the formation of hydrogen bonds between the three molecules and the nitrogen atom in the phosphorous-doped nitrogenous porous carbon material surface and (ii) the high surface area of the phosphorous-doped nitrogenous porous carbon material, which promotes the electrochemical signals. The effect of the pH was also studied (Figure 5D), revealing a shift of the oxidation peak potentials toward more negative values when the pH increases, indicating that a proton transfer accompanies the oxidation of the analytes. The optimization of the sensor design resulted in linear response ranges of 20–250 μM for ascorbic acid, 10–480 μM for DA, and 25–2500 μM for UA, with LODs of 4.25 μM, 0.86 μM, and 0.20 μM, respectively.

#### 4.1.3. Grain-Derived Biomass

Grains, such as rice, wheat, corn, and barley, are rich in carbohydrates and other organic compounds, making them suitable for developing electrodes and sensing platforms. Grain-derived biomass can be processed and treated to create carbon-based materials with desirable electrochemical properties [53,54,55,56]. Gissawong et al. [57] developed a novel, highly sensitive sensor for the antibiotic ciprofloxacin using DPV analysis. The glassy carbon electrode was modified by preparing a mixture of gold nanoparticles with waste coffee ground activated carbon and combined with the supramolecular solvent, which increased the electrochemical response of the nanoparticles. The calibration plot of the antibiotic exhibited a linear response in the range of 0.0005–0.025 µM with an impressive detection limit of 0.0002 µM. The sensor’s heightened sensitivity was attributed to the significantly increased electroactive area and conductivity resulting from the combination of waste coffee ground activated carbon and gold nanoparticles. The prepared sensor was successfully applied to determine ciprofloxacin in milk samples, achieving satisfactory recoveries ranging from 78.6 to 110.2%. On the other hand, Dinh Luyen et al. [58] synthesized a composite made of zeolite imidazolate framework-11 (ZIF-11) and activated carbon derived from rice husks and drop-casted it on the glassy carbon surface for sensing the biocide triclosan. The active carbon was prepared by carbonizing rice husk at 400 °C for 4 h; then, it was activated with activated carbon, KOH, and water under sonication conditions. Finally, the mixture was pyrolyzed at 750 °C for 1 h under a nitrogen atmosphere. The catalyst showed a high and homogeneous surface area and a high activity towards triclosan sensing. A linear range between 0.1–8 µM and an LOD of 0.076 µM were achieved.

#### 4.1.4. Seed-Derived Biomass

**Figure 6:**
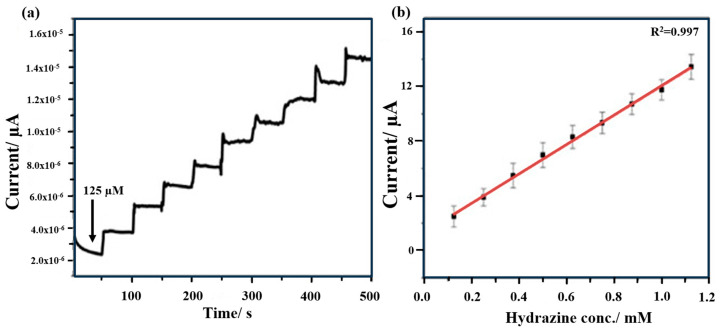
(**a**) Amperometric response of the carbon quantum dot-modified glassy carbon electrode towards sequential addition of hydrazine at 0.65 V vs. Ag/AgCl in 0.1 M phosphate buffer solution; (**b**) calibration curve representing the response of electrodes (N = 3) [65].

Seed-derived biomass-based electrochemical sensors have shown promising results in various applications. They have been utilized to detect environmental pollutants, making them relevant in environmental monitoring [59,60,61,62,63,64]. Regarding this, Sha et al. [65] presented a highly sensitive and selective sensor based on carbon quantum dots for the electrochemical determination of hydrazine. The catalyst was fabricated using chia seed as a natural and cost-effective precursor through a single-step pyrolysis process and subsequently employed for the modification of glassy carbon electrodes by drop-casting. The synthesized carbon quantum dots displayed a quasi-spherical morphology with a size distribution ranging from 2 to 6 nm and numerous surface functional groups. Amperometric measurements of the carbon quantum dot-based electrode showed a fast response towards hydrazine oxidation in the 125–1125 µM range at the potential 0.65 V under hydrodynamic conditions (Figure 6a). A good linearity between the concentration and the recorded current was observed (Figure 6b), resulting in a sensitivity of 151.5 µA mM^−1^ cm^−2^ and an LOD of 39.7 µM. Utilizing chia-seed-derived carbon quantum dots as the catalyst material offers an environmentally friendly and economical approach for the sensitive detection of hydrazine.

**Figure 7:**
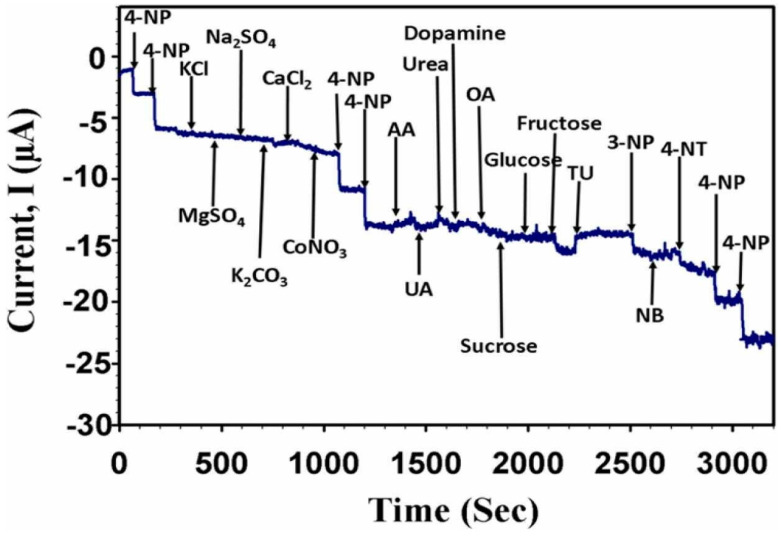
Selectivity study of glassy carbon electrode modified with gold nanoparticles/reduced graphene oxide with date-seed-derived biomass-derived activated carbon upon the injection of 100 µM 4-nitrophenol and five times (500 µM) higher concentrations of KCl, MgSO_4_, Na_2_SO_4_, K_2_CO_3_, CaCl_2_, CoNO_3_, urea, oxalic acid (OA), galactose, glucose, sucrose, and fructose, and three times (300 µM) higher concentrations of ascorbic acid, and dopamine, and similar (100 µM) concentrations of uric acid, thiourea (TU), 3-nitrophenol (3-NP), nitrobenzene (NB), and 4-nitrotoluene (4-NT) interfering chemicals in 0.1 M phosphate buffer solution (pH = 7.0) at a working potential of −0.6 V [66].

Likewise, an efficient electrochemical sensor for 4-nitrophenol utilizing a combination of gold nanoparticles/reduced graphene oxide with date-seed-derived biomass-derived activated carbon was successfully developed by Harraz et al. [66]. The synthesized catalyst demonstrated abundant active centers with enhanced electrochemical reductive activity, promoting the adsorption and diffusion of 4-nitrophenol molecules onto the active surface of the electrode. This facilitated superior electrochemical sensing capabilities. Notably, the sensor exhibited a remarkably low LOD of 0.36 µM and a high sensitivity of 0.03436 µA µM^−1^ within a wide linear dynamic range of 18–592 µM. Moreover, the modified electrode exhibited excellent selectivity in the presence of various common interfering compounds, such as ascorbic acid, UA, urea, glucose, and fructose, among many others (Figure 7). A list with more recent studies of herbaceous biomass and derivates for electrochemical sensing is listed in Table 3. As can be seen, different biomass-derived materials, such as fruit peels and seeds, have been used for sensing several groups of analytes, including heavy metals, dyes, and pharmaceutical-related compounds. Furthermore, techniques such as DPV, anodic stripping differential-pulse voltammetry (ASDPV), amperometry (Amp), and differential-pulse adsorptive stripping voltammetry (DPAdSV) are the most employed for sensing using biomass-derived carbon-based materials.

### 4.2. Woody Biomass and Derivates

**Figure 8:**
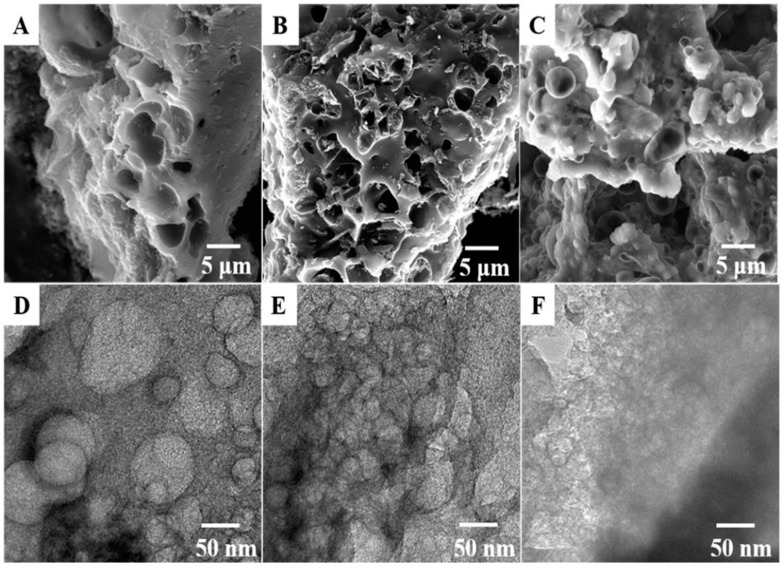
Scanning electron microscope images (**A**–**C**) and transmission electron microscopy images (**D**–**F**) of LRPC-800, N&P/LRPC-800-1, and N&P/LRPC-800-2, respectively [95].

Lignocellulosic-derived materials represent an abundant and cost-effective source of waste generated from agricultural, forestry, and industrial activities. This diverse group of materials comprises various components, such as coniferous or deciduous stems, branches, foliage, bark, chips, lumps, pellets, briquettes, sawdust, and sawmill, which possess distinct proportions of cellulose, hemicellulose, and lignin. These lignocellulosic materials have garnered attention for effectively removing contaminants and immobilizing heavy metals, owing to their interesting sorption properties and great cation exchange capacity. Despite these advantageous characteristics, these materials have not been extensively explored as potential electrode modifiers in electrochemical applications, despite their abundance and excellent properties. In light of the sustainable and renewable nature of lignocellulosic-derived materials, further research and investigation into their potential as electrode modifiers are warranted [90,91,92,93]. Similarly, a biosourced composite for the electrochemical sensing of the pesticide carbendazim was prepared by Fozing Mekeuo et al. [94] using *Ayous* (*Triphochiton Scheroxylon*) sawdust, an abundant waste from the wood industry, grafted by maleic anhydride. The resulting material was then mixed with carbon nanotubes and utilized for modifying glassy carbon electrodes by drop-coating. The effect of the amount of *Ayous* sawdust in the composite on the electrochemical sensing of carbendazim was evaluated. It was observed that increasing the percentage of modified lignocellulosic materials in the composite improved the sensor’s response by up to 4.8%. However, subsequent increases in the amount of *Ayous* sawdust beyond this point resulted in a progressive decrease in current intensities (ranging from 9 to 20%). This decrease was attributed to the insulating nature of *Ayous* sawdust, which hindered the electron transfer on the electrode. Under the optimized conditions, the developed sensor exhibited a sensitivity of 2.62 ± 0.08 µA M^−1^ and a detection limit of 0.04 µM. On the other hand, Lu et al. [95] prepared an N and P double-doped biomass pyrolytic carbon material for the electrochemical sensing of the flavonoids baicalein and luteolin. The authors designed the catalyst by pyrolyzing lotus roots as a source of biomass, KOH, as an activator, and ammonium polyphosphate, as a source of N and P. The mixtures were heated at 800 °C for 2 h in a N_2_ atmosphere. The amount of APP was systematically tested, and the resulting catalyst (N&P/LRPC-800-1) was compared with the materials obtained without APP and with a lower amount of ammonium polyphosphate (LRPC-800 and N&P/LRPC-800-2, respectively). Scanning electron microscopy analysis (Figure 8A–C) revealed that N&P/LRPC-800-1 possessed a more porous structure, which was further supported by the transmission electron microscopy analysis (Figure 8D–F) showing a more developed pore structure inside. This confirms that an appropriate amount of ammonium polyphosphate doping effectively improves the specific area of the carbon materials, while an excess of ammonium polyphosphate content could sharply reduce it. Under the optimal conditions, the system achieved an LOD of 0.0078 µM and 0.0076 µM for BA and LU, respectively. Both studies highlight the potential of utilizing biomass-derived materials for developing efficient and sensitive electrochemical sensors, providing valuable insights for further advancements in the field of electrochemical sensing.

A low-cost carbon paste electrode modified with an aminoalcohol-functionalized palm oil fiber for 2-nitrophenol electrochemical sensing was prepared by Deussi Ngaha et al. [96]. The catalyst was prepared by the chemical grafting of triethanolamine onto the surface of an alkali material. The constructed electrode demonstrated high sensitivity for 2-nitrophenol electrochemical detection, covering a wide linear range of 4–50 µM and exhibiting an LOD of 1.26 µM. Additionally, the prepared electrode showed excellent reproducibility and stability, with standard deviations of 2.277% (N = 5) and 5.268% (N = 5), respectively. The practical application of the sensor was evaluated by detecting 2-nitrophenol in lake, spring, and tap water samples using the standard addition method. Analysis observed recovery rates of 99.02%, 99.52%, and 99.95%, respectively, signifying the successful application of the proposed method for determining this compound in real environmental samples. Using aminoalcohol-functionalized palm oil fiber as the electrode modifier offers a promising approach for sensitive and reliable electrochemical sensing of 2-nitrophenol. Several recent studies of woody biomass and derivates for electrochemical sensing are listed in Table 3. As can be seen, several catalysts have been developed using woody-derived materials, which have been mainly used for the electrochemical sensing of organic compounds.

### 4.3. Animal and Human Waste

Animal and human wastes are predominantly sourced from bones, meat meals, certain manures, and human dung. In the past, these wastes were widely employed as fertilizers; however, their utilization has gradually diminished over time due to agricultural and environmental regulations concerning pollution, health, odor, and other concerns. The use of animal and human waste as biomass for electrochemical sensing has gained attention as a sustainable and eco-friendly approach in recent years. These waste materials, traditionally considered by-products or pollutants, possess unique chemical compositions and properties that make them potential candidates for developing low-cost and efficient electrochemical sensors [97,98,99,100,101,102,103].

#### 4.3.1. Animal-Derived Waste

**Figure 9:**
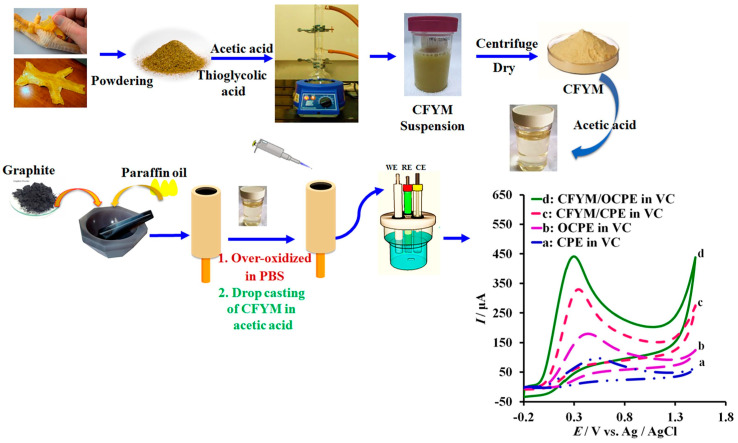
Schematic methodology for the determination of Vitamin C using a yellow membrane of chicken feet-derived waste [104].

Animal-derived waste represents a valuable and often overlooked resource for developing eco-friendly and cost-effective electrochemical sensors. Razmi et al. [104] developed a cost-effective and sensitive sensor to detect vitamin C by DPV. The authors used the yellow membrane of chicken feet as the base for the sensor. This membrane is a natural and porous substance composed of carbohydrates and a mix of specific amino acids, such as glycine and arginine. It also contains several functional groups such as -OH, -COOH, -C=O, and -NH_2_, which make it suitable for adsorbent and preconcentration. The authors demonstrated that the over-oxidized carbon paste electrode modified with the membrane (Figure 9) exhibited a remarkable improvement in the electron transfer kinetics for Vitamin C by lowering the anodic over-potential and increasing the anodic peak current. The sensor performance achieved a low LOD of 5.96 µM, a wide linear range of 9.9–280.5 µM, and a good sensitivity of 2.1969 µA/µmol dm^−3^.

**Figure 10:**
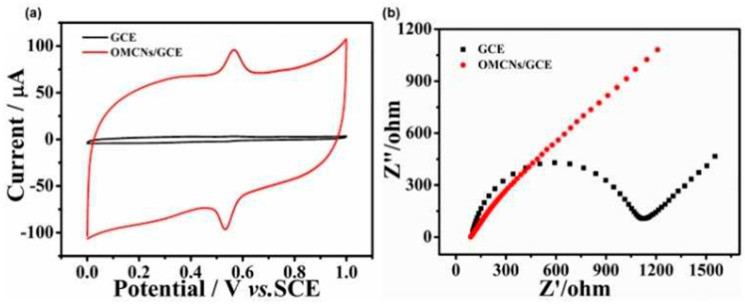
(**a**) Cyclic voltammograms of 25 μM malachite green in 0.05 M H_2_SO_4_ on the ordered mesoporous carbon nanofiber arrays on glassy carbon (red line) and glassy carbon (black line), with the scan rate = 100 mVs^−1^ and (**b**) Nyquist plots corresponding to the glassy carbon and ordered mesoporous carbon nanofiber arrays on glassy carbon surface in 5 mM Fe(CN)^−3^/^−4^ +0.1 M KCl [85].

Yang et al. [85] demonstrated the utilization of a natural crab shell as a template to synthesize ordered mesoporous carbon nanofiber arrays to prepare an electrochemical sensor for the dye malachite green by DPV. Crab shell has been used as a biological template for generating hierarchical structures, allowing the recycling of environmental waste to produce value-added materials. Moreover, the cost of the obtained materials is considerably lower than that of others from commercial sources. The synthesized material presented ordered mesoporous carbon arrays with a high BET surface of 1200 m^2^g^−1^, which provides a high surface area. The electrochemical characterization (Figure 10a) showed that the synthesized ordered mesoporous carbon nanofiber arrays presented reversible redox peaks for the malachite green process, with a difference between the anodic peak and the cathodic peak of only 40 mV, attributed to the good electrochemical properties of the catalyst. The analysis of electrochemical impedance spectroscopy (Figure 10b) confirmed the rapid electron transfer of the prepared material compared to the bare glassy carbon electrode. The sensor showed a good response for the dye sensing, showing a wide linear range from 0.1 to 22.1 µM and a low detection limit of 0.05 µM. This outstanding performance makes the prepared catalyst a promising candidate for the sensitive and efficient detection of malachite green in various environmental and analytical applications.

#### 4.3.2. Human-Derived Waste

**Figure 11:**
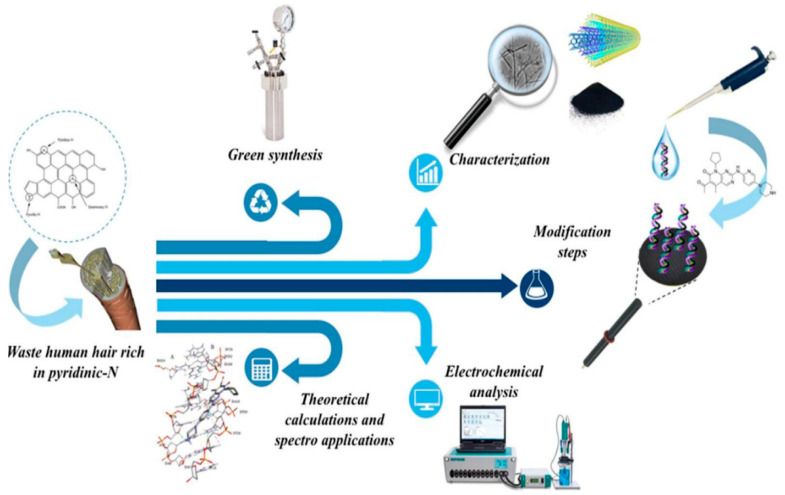
Schematic methodology to electrochemical monitoring of Palbociclib–DNA interaction using human hair waste [105].

Human-derived waste-based electrochemical sensors have the potential to revolutionize healthcare and environmental monitoring by offering non-invasive, real-time, and cost-effective solutions. Bilge et al. [105] prepared a carbon material from human hair waste by hydrothermal carbonization and KOH activation. Human hair has attracted much attention due to its potential to obtain heteroatom-doped and porous carbon materials due to hair fibers’ hierarchical structures with rich nitrogen and sulfur content. The authors mixed the prepared hair with amine-functionalized multi-walled carbon nanotubes and supported it on a glassy carbon electrode for deoxyribonucleic acid (DNA) biosensor experiments (Figure 11). The prepared biosensor was utilized to study the dsDNA–Palbociclib (PLB) interaction to assess the possibility that PLB produces conformational changes in DNA structure and/or oxidative damage. The glassy carbon modified with hair with amine-functionalized multi-walled carbon nanotubes was an efficient biosensing platform for dsDNA immobilization and nanobiosensor fabrication due to the sensitivity being significantly increased compared to bare glassy carbon electrode. A linear dependence was obtained in the 0.4–10 µM range.

A list with more recent studies of animal and human waste for electrochemical sensing is listed in Table 3. Bovine bones, crab-derived waste, eggshell membrane, and pig blood have been used to develop a wide variety of target molecules, including H_2_O_2_, heavy metals, and organic compounds.

### 4.4. Other Biomass Sources

#### 4.4.1. Fungi

Yin et al. [106] modified a glassy carbon electrode with a composite made of sulfur-doped graphene (SG), carboxylated carbon nanotube (COOH-CNT), and yeast for the electrochemical detection of lead ions. The composite was prepared using a simple hydrothermal method. Yeast is one of the most-studied eukaryotic microorganisms for removing large numbers of heavy metals from environmental samples. This microorganism, when treated under alkali hydrothermal conditions, modifies cells with functional groups such as amines, hydroxyl, and carboxylate groups, which are available to interact with other molecules of interest and construct different architectures. The prepared composite exhibited a very high response at detecting low concentrations of lead ions, achieving a linear range between 4.9 × 10^−9^ and 4.910^−17^ µM and an LOD of 2.61 × 10^−17^ µM, being capable of detecting Pb^2+^ levels in blood with a satisfactory recovery rate. The authors attributed the high sensing performance to the double-layer carbon structure formed by the combination of sulfur-doped graphene and carboxylated carbon nanotubes.

#### 4.4.2. Aquatic Biomass

**Figure 12:**
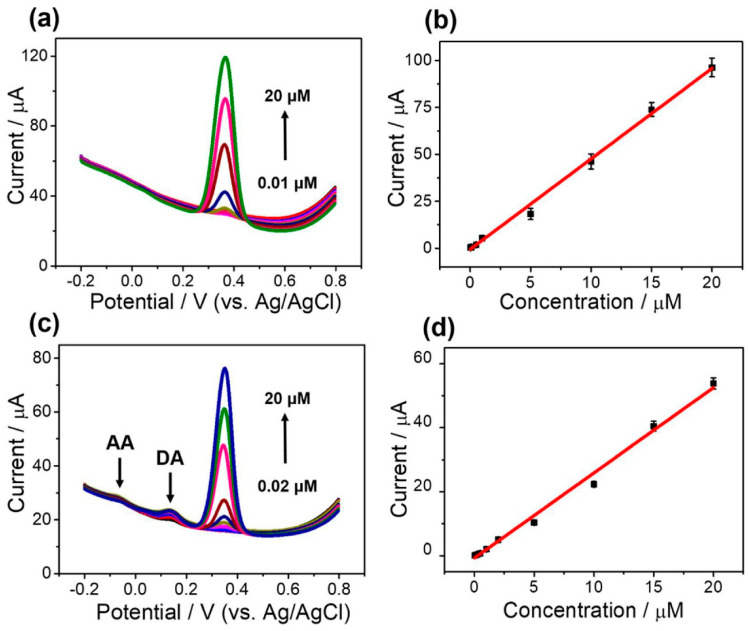
Determination of AC in 0.1 M phosphate buffer solution (pH = 7.4) using glassy carbon modified with the catalyst obtained with consecutive activations. (**a**) DPV plots and (**b**) corresponding linear calibration plots of the result. The range of concentration of acetaminophen was from 0.01 µM to 20 µM. The determination of acetaminophen in 0.1 M phosphate buffer solution (pH = 7.4) using glassy carbon modified with the catalyst obtained with consecutive activations with 100 µM of ascorbic acid and 1 µM of dopamine. (**c**) DPV plots and (**d**) corresponding linear calibration plots of the result. The range of concentration of AC was from 0.02 µM to 20 µM [16].

Aquatic biomass represents a promising and versatile material for biosensor development, offering a sustainable and cost-effective solution for various sensing applications in environmental, biomedical, and industrial fields [16,107,108]. Kim et al. [16] reported a novel two-step activation of biomass-derived carbon for the electrochemical sensing of acetaminophen, using kelp powder activated using (i) ZnCl_2_, (ii) KOH, and (iii) double-step activation using both as activator agents. Kelp, a seaweed and natural source of glutamate, is also frequently used in East Asian nations as a food additive. Moreover, being abundant in the ocean around the world and the relatively small human consumption leaves an abundant amount of unused seaweed, which decreases the costs for its use as a material. The characterization of activated materials showed that the activation increases the BET surface area, pore volume, and Barrett, Joyner, Halenda (BJH) pore size, achieving higher values using consecutive activations. The determination of acetaminophen was studied by DPV using a glassy carbon modified with the catalyst obtained with consecutive activations (Figure 12a,b). A linear response of the current with the CA concentration from 0.01 to 20 µM was observed; no changes were observed when the analysis was performed in the presence of interferents such as ascorbic acid and dopamine (Figure 12c,d). The LOD of the prepared sensor was 0.005 µM.

#### 4.4.3. Industrial Biomass Wastes (Semi-Biomass)

**Figure 13:**
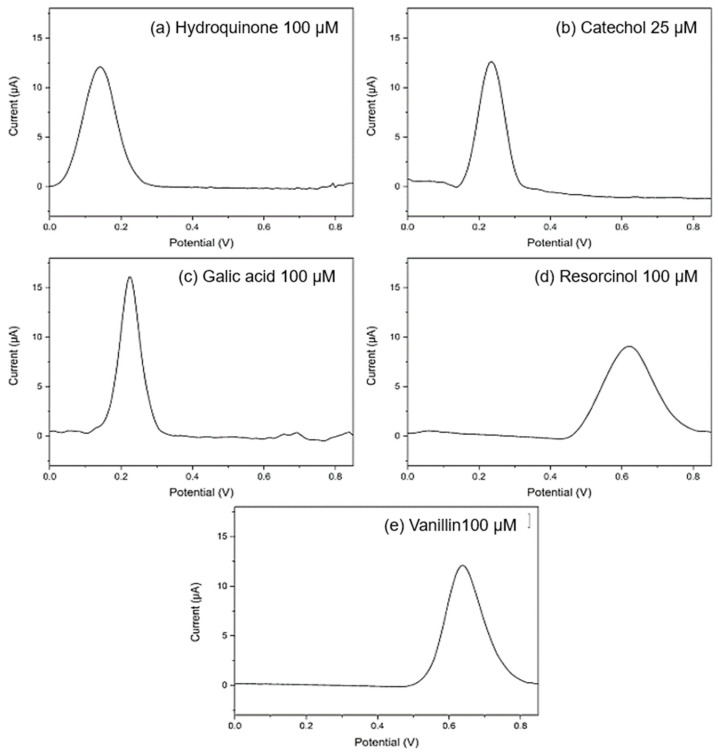
Differential pulse voltammograms recorded in 0.1 M acetic buffer pH 4.75 at the prepared catalyst for (**a**) 0.1 mM hydroquinone; (**b**) 0.025 mM catechol; (**c**) 0.1 mM gallic acid; (**d**) 0.1 mM resorcinol; (**e**) 0.1 mM vanillin; pulse amplitude 90 mV, pulse width 60 ms, and scan rate 30 mVs^−1^ [109].

The recycling of carbonaceous materials obtained from different industrial wastes has received increasing attention for its use in many areas. In particular, the similarities between some treated recycled carbonaceous materials with carbon black, such as amorphous carbon, containing minimal amounts of oxygen, nitrogen, and hydrogen, represent a low-cost alternative to other carbon-based materials in electrochemical sensing and energetics. In this context, Sfragano et al. [109] proposed an innovative carbon paste electrode composed of biochar derived from biological sludge from municipal and industrial wastewater treatment plants for sensing phenolic compounds. The catalyst was prepared via the pyrolysis of a mixture of waste woody biomass and biological sludge under a N_2_ atmosphere at 850 °C for 60 min. The electrode for the sensing was prepared by mixing the obtained product after the pyrolysis with graphite powder and mineral oil. The catalyst obtained possesses a mesoporous structure. DPV scans of different polyphenolic compounds were performed using the fabricated sensor (Figure 13). The results showed that the electrode was able to distinguish among different phenol structures and from various concentrations, achieving linear behavior in the range of 10–150 µM.

Given these results and those shown in Table 3, there is no doubt that new approaches will be studied using industrial wastes.

## 5. Conclusions and Perspectives

This article offers a brief overview of the recent developments in using biomass-based carbon materials for the electrochemical sensing of different substances. The article discusses the methods used in creating these materials and some modifications to improve their structure, enhancing their ability to sense electrochemical reactions. Additionally, the article touches on various characterization techniques, focusing on the crucial electrochemical parameters essential in creating highly active and stable sensors.

The presence of specific functional groups on the surface of the treated material plays a crucial role in determining its affinity towards the targeted molecule of interest. The synthesis procedure offers an opportunity to deliberately modulate the presence of these functional groups, allowing for the development of intelligent and tailored structures to suit specific sensing requirements. While material stability can sometimes pose a limitation for real-world, large-scale implementation, the advantages of using low-cost and abundant materials can outweigh concerns about stability. Moreover, some synthesis procedures are straightforward and do not require significant financial investment, making them accessible for broader adoption.

Reducing industrial waste and implementing recycling practices are crucial steps in fostering a more sustainable environment. By minimizing waste generation, we contribute to environmental preservation and realize cost reductions in obtaining materials. In line with this goal, the concept of a circular economy emerges as a promising approach, advocating for using biomass-derived materials across various applications, including the development of carbon-based electrodes. Their versatility and adaptability make them attractive candidates for multiple purposes, particularly in designing efficient and cost-effective carbon-based electrodes. Integrating biomass-derived materials in electrode development and other innovative solutions holds great promise for advancing sustainable practices, reducing waste, and fostering a greener future. By aligning our efforts with this paradigm, we can make significant strides toward a more environmentally conscious and resource-efficient society.

## Figures and Tables

**Table 1:** Range of chemical composition of different biomass classifications [5].

Biomass Group	C %	O %	H %	S %	N %	Volatile Matter %	Fixed Carbon %	Moisture %	Ash %
Herbaceous biomass	42–58	34–49	3–9	<1–1	<1–3	41–77	9–35	4–48	1–19
Woody biomass	49–57	32–45	5–10	<1–1	<1–1	30–80	6–25	5–63	1–8
Animal and human waste	57–61	21–25	7–8	1–2	6–12	43–62	12–13	3–9	23–34
Aquatic biomass	27–43	34–46	4–6	1–3	1–3	42–53	22–33	8–14	11–38

**Table 2:** Advantages and disadvantages of different methods used for the synthesis of BM-derived carbon materials.

Method	Advantages	Disadvantages
Pyrolysis	Tunable properties User friendly	Slow rate of production Significant air pollution Difficult to know the mechanism Low variety of functional groups on the surface material
Hydrothermal carbonization	Less toxicity Tunable morphology Abundant heteroatoms can be doped	Large size of carbon particles Sealed vessel required
Ionothermal carbonization	No need for the utilization of high temperatures and sealed vessels Abundant heteroatoms can be doped IL can be recovered and reused	Expensive
Template-assisted method	Highly porous material	Difficult to remove the template Low stability of the template at high temperatures Multi-step process More expensive

**Table 3:** List of recent research on biomass-derived carbon-based materials for electrochemical sensing.

Sensing Platform	Biomass-Derived	Analyte	Technique	LR (μM)	LOD (μM)	Ref.
HVE-Na_2_CO_3_/CPE	*Hordeum vulgare* dust	Cd^2+^, Pb^2+^ and Hg^2+^	ASDPV	-	1.82 (Cd^2+^), 0.000691 (Pb^2+^) and 0.000237 (Hg^2+^)	[67]
BPBC-MWCNT/GCE	Banana peel	Baicalein	DPV	0.0040–100.0	0.00133	[68]
CNANAs/GCE	Shaddock peel waste	H_2_O_2_	Amp	5–1760	3.53	[69]
N,P-MMC	Okra	H_2_O_2_	Amp	100–10,000 and 20,000–200,000	6.8	[70]
N-NPC-Nafion/GCE	Almond shells	Pb(II)	ASDPV	0.0097–0.5797	0.0034	[71]
CDs-Cu_2_O/CuOGCE	Reeds	Hydrazine	Amp	0.99 μM to 5903	0.024	[72]
CP-OM-COOH	Grapefruit peel	Cu(II)	DPV	0.2362–1.0236	0.0394	[73]
CDs/SPCE	Orange peel waste	Nitrobenzene	DPV	0.1–2000	0.013	[74]
CPME-ACfB 300	Spent coffee grounds	Pb(II)	DPAdSV	0.128–2.44	0.0045	[75]
CH-CPE	Coffee husks	Methylene Blue	SWV	1–125	3	[76]
CuCo_2_O_4_@BC	*Lactuca sativa* L. *var. Ramosa*	Tryptophan	DPV	0.01–1 and 1–40	0.003	[77]
PC_900_/GCE	*Liquidambar formosana* tree leaves	3-nitroaniline and 4-nitroaniline	DPV	0.2–115.6 (3-nitroaniline) 0.5–120 (4-nitroaniline)	0.0551 (3-nitroaniline) and 0.0326 (4-nitroaniline)	[78]
CDs/GCE	*Eclipta Alba* leaves	Morin	DPV	50–350	1.42 × 10^−7^	[79]
N,P-CQD	*Banana flower bract (Musa acuminata)*	Dopamine	DPV	6–100	∼0.0005	[30]
BG-CNPs/GC	Cherry (*Physalis) peruviana*) husks	H_2_O_2_	Amp	10–220	1.6	[80]
BC/Co_3_O_4_/FeCo_2_O_4_/GCE	Pinecones	Dopamine, acetaminophen, and xanthine	DPV	0.1–250 (Dopamine) 0.1–220 (Acetaminophen), and 0.5–280 (Xantine)	0.04587 (Dopamine), 0.02886 (Acetaminophen), and 0.1209 (Xantine)	[81]
PANI/MWCNT/Cotton Yarns	Cotton yarns	Urea	Amp	0.001–1000	0.001	[82]
NSPAC/GCE	Chinese fir sawdust	Dopamine and uric acid	DPV	0.2–100.0 (Dopamine), 0.2–50.0 (Uric acid)	0.1 (for Dopamine and Uric acid)	[83]
GrRAC-70%	Cork powder	Caffeine	DPV	2.5–1000	2.94	[84]
OMCNs	Crab shell	Malachite green	DPV	0.1–22.1	0.05	[85]
ESM-AC	Egg shell membrane	Dopamine	DPV	100–10,000	0.26	[86]
GCE/NHAPP0.5-CA-β-CD	Bovine bones	Pb(II)	DPASV	0.02–0.20	0.000506	[87]
Fe-BCs	Pig blood	H_2_O_2_	Amp	0.1–2000	0.046	[88]
r-rGO/GCE	Waste from powder juice industry	Paracetamol	DPV	60–500	0.28	[89]

## Data Availability

Data are contained within the article.
